# *In vitro* phenotypic susceptibility of HIV-1 non-group M to CCR5 inhibitor (maraviroc): TROPI-CO study

**DOI:** 10.1128/spectrum.03895-23

**Published:** 2024-05-29

**Authors:** Ségolène Gracias, Ikrame El Yaalaoui, Benoît Visseaux, Charlotte Charpentier, Diane Descamps, Charlène Martin, Fanny Lermechain, Jean-Christophe Plantier, Elodie Alessandri-Gradt

**Affiliations:** 1 Univ Rouen Normandie, Université de Caen Normandie, INSERM, Normandie Univ, DYNAMICURE UMR 1311, CHU Rouen, Department of virology, Rouen, France; 2 Service de virologie, IAME, INSERM, UMR 1137, AP-HP, Hôpital Bichat-Claude Bernard, Université Paris Cité, Paris, France; Kumamoto Daigaku, Kumamoto, Japan

**Keywords:** human immunodeficiency virus, genetic diversity, phenotypic susceptibility, maraviroc

## Abstract

The susceptibility of genetically divergent HIV-1 strains (HIV-1 non-M) from groups O, N, and P to the CCR5 co-receptor antagonist, maraviroc (MVC), was investigated among a large panel of 45 clinical strains, representative of the viral genetic diversity. The results were compared to the reference strains of HIV-1 group M (HIV-1/M) with known tropism. Among the non-M strains, a wide range of phenotypic susceptibilities to MVC were observed. The large majority of HIV-1/O strains (40/42) displayed a high susceptibility to MVC, with median and mean IC_50_ values of 1.23 and 1.33 nM, respectively, similar to the HIV-1/M R5 strain (1.89 nM). However, the two remaining HIV-1/O strains exhibited a lower susceptibility (IC_50_ at 482 and 496 nM), in accordance with their dual/mixed (DM) tropism. Interestingly, the two HIV-1/N strains demonstrated varying susceptibility patterns, despite always having relatively low IC_50_ values (2.87 and 47.5 nM). This emphasized the complexity of determining susceptibility solely based on IC_50_ values. Our study examined the susceptibility of all HIV-1 non-M groups to MVC and correlated these findings with virus tropism (X4, R5, or DM). The results confirm the critical significance of tropism determination before initiating MVC treatment in patients infected with HIV-1 non-M. Furthermore, we advocate for the consideration of additional parameters, such as the slope of inhibition curves, to provide a more thorough characterization of phenotypic susceptibility profiles.

**IMPORTANCE:**

Unlike HIV-1 group M, the scarcity of studies on HIV-1 non-M groups (O, N, and P) presents challenges in understanding their susceptibility to antiretroviral treatments, particularly due to their natural resistance to non-nucleoside reverse transcriptase inhibitors. The TROPI-CO study logically complements our prior investigations into integrase inhibitors and anti-gp120 efficacy. The largest panel of 45 non-M strains existing so far yielded valuable results on maraviroc (MVC) susceptibility. The significant variations in MVC IC50 reveal a spectrum of susceptibilities, with most strains displaying R5 tropism. Notably, the absence of MVC-resistant strains suggests a potential therapeutic avenue. The study also employs a robust novel cell-based phenotropism assay and identifies distinct groups of susceptibilities based on inhibition curve slopes. Our findings emphasize the importance of determining tropism before initiating MVC and provide crucial insights for selecting effective therapeutic strategies in the delicate context of HIV-1 non-M infections.

## INTRODUCTION

The high genetic diversity of HIV-1 has led to the current classification of four subgroups (M, N, O, and P). Only HIV-1 group M (HIV-1/M) has driven the worldwide pandemic (1). HIV-1 non-M group (HIV-1 non-M) is endemic in west-central Africa. HIV-1 group O (HIV-1/O) is subdivided into two main subgroups (T and H) (2) and is particularly found in Cameroon, where it represents 0.6%–1% of HIV diagnoses. Only sporadic cases have been detected in other continents (3). HIV-1 group N (HIV-1/N) and HIV-1 group P (HIV-1/P) are even rarer, with, respectively, fewer than 20 and 2 reported cases of infection (3, 4). The French National Survey Network (RES-O) takes a census of all HIV-1/non-M infections in France and has identified more than 140 cases of HIV-1/O infections, one primary HIV-1/N infection, and one HIV-1/P infection, the most recent prototype strain of group P known to date.

The specific genetic diversity of the HIV-1 non-M viruses has known impacts on the antiretroviral treatments’ susceptibility. It has been previously demonstrated that the HIV-1/O are naturally resistant to the non-nucleoside reverse transcriptase inhibitors, particularly due to the Y181C resistance mutation present in 75% of the group O strains. The other antiretroviral agents have various *in vitro* efficacies, demonstrating the need for studying large panels of strains for HIV-1 non-M (5–7). The envelope region of HIV-1 non-M has a particularly high level of genetic diversity in comparison with HIV-1/M envelope amino acid sequences (8). The genetic sequence of the envelope V3 loop domain is commonly used as a predictor of the coreceptor use: CXCR4, CCR5, or both, defining the virus tropism, usually called X4, R5, or dual/mixed (DM), respectively (9–11). Maraviroc (MVC) is the only CCR5 competitive antagonist commercialized since 2007 for patients with treatment failure (12). Because of its mechanism of action, this molecule is only effective on R5 viruses (13). Even if R5 tropism is the most frequent in the population (14), a predictive assay of virus tropism must always be performed before MVC initiation (11). To date, MVC is an alternative therapeutic strategy for patients previously treated and who may have acquired multiple resistances to diverse antiretrovirals. The choice of the appropriate antiretroviral combination is even more important when dealing with drug-resistant viruses or patients presenting contraindications or allergies to medications that further limit the availability of fully effective medications. In this context, MVC could be a choice molecule to achieve a fully active therapeutic combination for patients with additional resistance mutations. Due to less than 40% similarity between group O and M V3 loop sequences, it has previously been demonstrated that common HIV-1/M genotypic rules have failed to correctly predict the HIV-1 non-M tropism. There is only one study reporting the absence of correlation between five genotypic tools and *in vitro* phenotypic tropism prediction in 18 group O strains (11).

Considering the absence of HIV-1 non-M approved tropism assay prior to MVC administration and the lack of *in vitro* data about MVC efficiency on HIV-1 non-M strains, the aim of this study was to define the phenotypic susceptibility of a large HIV-1 non-M panel (45 strains) to MVC and correlate these findings with the phenotypic tropism obtained by an in-house fluorescent cell assay.

## RESULTS

**Fig 1:**
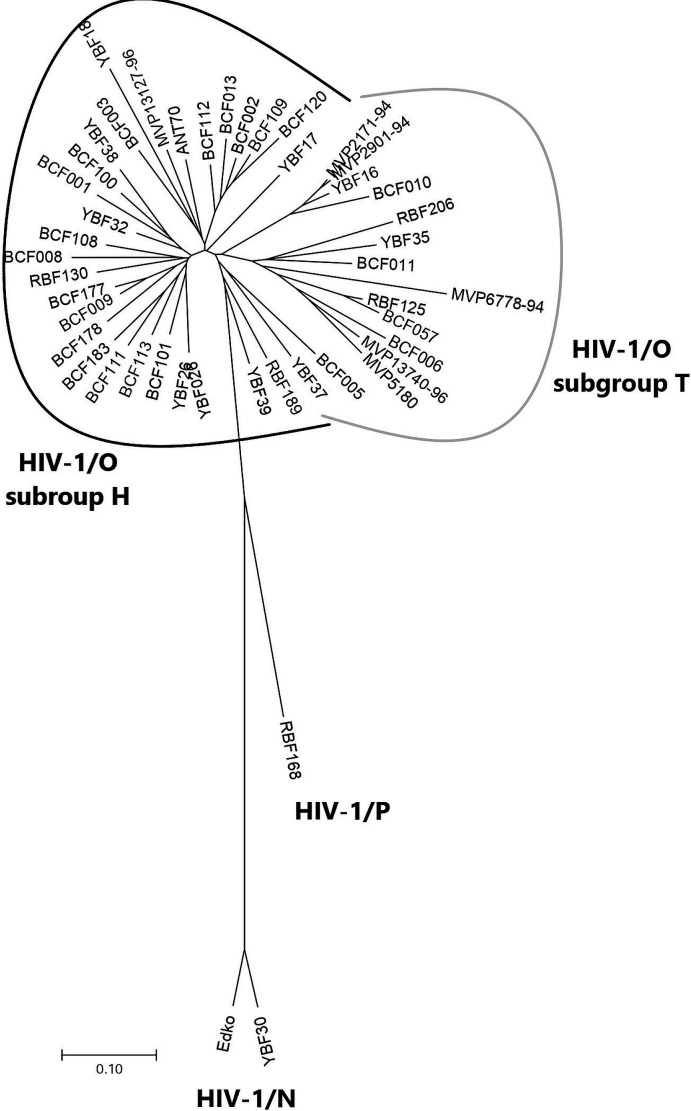
Phylogenetic tree of the 42 HIV-1/O, 2 HIV-1/N, and 1 HIV-1/P, V3 sequences from the strains included in this study.

We analyzed 45 HIV-1 non-M clinical isolates: 42 HIV-1/O, 2 HIV-1/N, and 1 HIV-1/P, representative of HIV-1 genetic diversity as demonstrated with a phylogenetic tree based on V3 sequences (Fig. 1), compared to 4 HIV-1/M control strains.

The HIV-1/M R5 strain (ARP1102) entry was efficiently blocked by MVC with an IC_50_ at 1.89 nM and maximal plateau of inhibition (MPI) at 90.3%, whereas X4 strains (BRU-HXB2, ARP1196, and JR001) were all resistant (IC_50_ > 1,000 nM) and MPI at 0%, in accordance with what was expected for these reference strains (Table 1). The HIV-1/M DM tropism strain (ARP1129) was also resistant to MVC (IC_50_ > 1,000 nM) and MPI at 6.9%. These five strains were also used to validate our tropism determination method, as the tropism was already known.

**TABLE 1:** Results of tropism IC_50_ and MPI for the reference strains of HIV-1/M

Reference strain	Tropism	IC_50_ (nM)	MPI (%)
ARP1102	R5	1.89	90.3
ARP1129	DM	>1,000	6.9
ARP1196	X4	>1,000	0
BRU-HXB2	X4	>1,000	0
JR001	X4	>1,000	0

**Fig 2:**
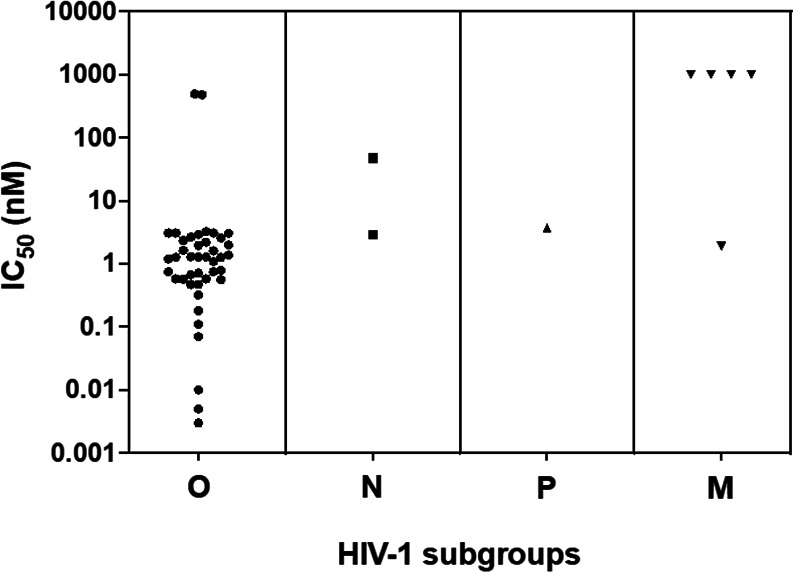
Comparison of HIV-1 non-M IC_50_ (nM) (42 HIV-1/O, 2 HIV-1/N, and 1 HIV-1/P strains). The strains with a decrease of less than 1,000 HIV GE/µL of viral load between the lowest and the highest concentration are considered as having a low susceptibility to MVC.

Among the 45 available HIV-1 non-M strains, 40 HIV-1/O were susceptible to MVC with IC_50_ between 0.003 (YBF37) and 3.22 nM (BCF100), with corresponding median and mean IC_50_ of 1.23 and 1.33 nM, respectively (Fig. 2). The mean MPI (min; max) was at 95.3% (85.5; 98.9) (Table 2). The mean IC_50_ of these 40 strains did not differ significantly from the susceptible group M strain. According to our phenotypic tropism assay, all the strains demonstrated an R5 tropism except one (YBF18), which displayed a DM tropism with high susceptibility to MVC: IC_50_ at 0.57 nM and 94.7% MPI.

**TABLE 2:** Results of tropism, IC_50_, MPI, and hill slope obtained for each control and clinical strain of the panel

Strain	Group	Subgroup	Tropism	IC_50_ (nM)	MPI (%)	Hill slope	GenBank no.
BCF006	O	T	DM	496	65.5	ND	
MVP5180	O	T	DM	482	29.6	ND	
YBF18	O	H	DM	0.57	94.7	3.71	
N1FR2011	N		R5	47.5	63.7	1.09	JN572926
RBF168	P		R5	3.68	88.3	0.91	GU111555
BCF100	O	H	R5	3.22	94.2	1.57	
BCF108	O	H	R5	3.1	85.5	2.15	
BCF101	O	H	R5	3.09	95.5	1.06	
MVP2901-94	O	T	R5	3.08	95	1.43	
BCF111	O	H	R5	3.05	93.4	1.52	
BCF112	O	H	R5	2.93	92.7	1.05	
YBF30	N		R5	2.87	94.3	1.1	AJ006022
BCF177	O	H	R5	2.66	90.6	1.57	
BCF010	O	T	R5	2.57	97.3	1.08	
BCF113	O	H	R5	2.34	94.2	2.23	
BCF057	O	T	R5	2.21	96.9	1.47	
MVP2171-94	O	T	R5	1.99	95.5	1.64	
BCF011	O	T	R5	1.96	94.2	1.14	
BCF001	O	H	R5	1.63	96.6	1.3	
BCF013	O	H	R5	1.61	97.9	1.83	
YBF32	O	H	R5	1.38	95.7	0.77	
RBF130	O	H	R5	1.3	96.4	1.21	
BCF003	O	H	R5	1.28	95	0.9	
RBF206	O	T	R5	1.28	97.3	0.68	
BCF120	O	H	R5	1.27	95.4	1.37	
YBF38	O	H	R5	1.26	93.9	2.12	
BCF009	O	H	R5	1.2	96.9	0.94	
ANT70	O	H	R5	1.09	95.6	1.89	
MVP13740-96	O	T	R5	0.79	95.1	0.56	
YBF16	O	T	R5	0.75	97.2	1.48	
BCF109	O	H	R5	0.75	96.1	1.16	
BCF005	O	T	R5	0.71	97.3	0.95	
MVP6778-94	O	T	R5	0.67	98.1	2.32	
MVP13127-96	O	H	R5	0.58	98	1.62	
BCF002	O	H	R5	0.58	98.9	0.81	
RBF189	O	H	R5	0.56	98.8	1.09	
BCF178	O	H	R5	0.47	98	1.3	
BCF183	O	H	R5	0.47	96.7	0.94	
RBF125	O	T	R5	0.32	97.7	0.62	
YBF35	O	T	R5	0.18	97.2	1.12	
YBF17	O	H	R5	0.11	98	1.43	
BCF008	O	H	R5	0.07	97.2	1.53	
YBF26	O	H	R5	0.01	89.9	0.7	
YBF39	O	H	R5	0.005	85.8	0.67	
YBF37	O	T	R5	0.003	90.8	0.73	

No strain of the non-M panel showed a resistant profile similar to the X4 group M reference (Fig. 2). However, the two remaining HIV-1/O strains expressed a very low MVC-sensitive phenotype (MVP5180 and BCF006), with IC_50_ and MPI at 482 nM; 30% and 496 nM; 66%, respectively. These two strains exhibited DM profiles on the phenotypic tropism assay (Table 2) and had IC_50_ statistically different (*P* = 0.016) from the M reference R5 strain.

Regarding the two HIV-1/N strains, the first revealed an MVC susceptibility with an IC_50_ and MPI at 2.87 nM and 94% (YBF30). The second (N1FR2011) was less susceptible to MVC with an IC_50_ at 47.5 nM and an MPI > 50% (63.7%), despite an R5 phenotypic tropism (Table 2).

The HIV-1/P strain (RBF168) showed an IC_50_ at 3.68 nM and MPI at 88%; this strain also showed an R5 phenotypic tropism.

**Fig 3:**
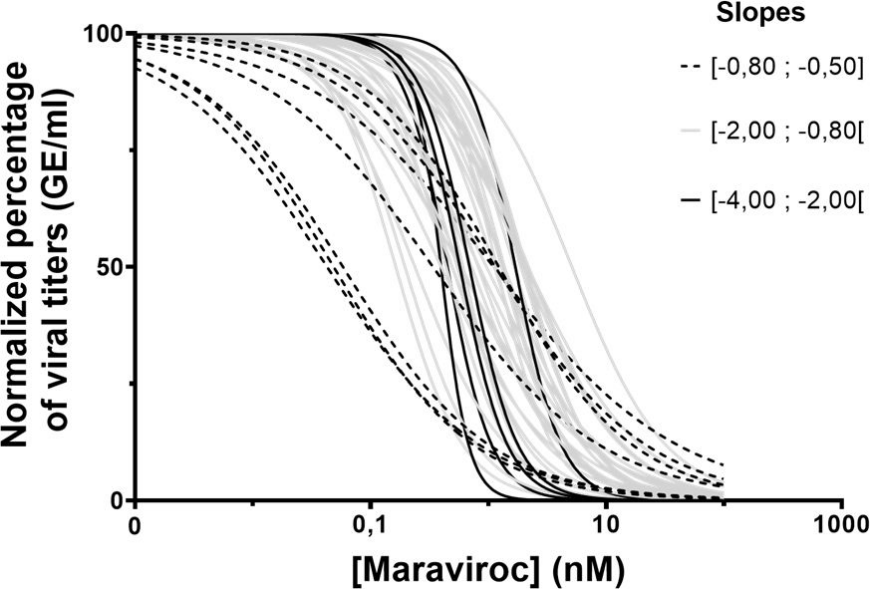
Susceptibility of 42 HIV-1/O expressed by normalized viral replication on maraviroc serial concentrations. Each curve depicts a different strain, and the different patterns are used to group the strains with similar slopes.

This wide range of various MVC susceptibilities among the non-M panel is represented in Fig. 2 and 3. We distinguished the strains with low MPI (<50%) expressing weak susceptibility (*N* = 1, MVP5180) and the strains with high susceptibility MPI (>70%) (*N* = 40), representing the majority of the panel, as well as the strains with intermediate susceptibility (50% < MPI < 70%) (two strains). Furthermore, we noticed, among the highly susceptible strains, different behaviors from the shape of the inhibition curve. In this analysis, the two DM HIV-1/O strains less sensitive were not included. Thus, three groups of susceptible strains could be identified (Fig. 3) from the hill-slope value of the inhibition curve. The group with the steeper slopes (−4; −2) included five HIV-1/O strains with median IC_50_ (min; max) of 0.79 (0.57; 31). This group also included the DM HIV-1/O YBF18 MVC-susceptible strain. The group with intermediate slopes between −2 and −0.80 was the most important, containing 31 strains (20 subtype H; 8 subtype T; 2 HIV-1/N; and 1 HIV-1/P). A wide range of IC_50_ was also observed in this group with a median IC_50_ (min; max) at 1.28 nM (0.07; 47.5). The last group with slopes close to 0 (−0.8; −0.5) comprised seven HIV-1/O with a corresponding median IC_50_ at 0.71 nM (0.03; 1.28).

**Fig 4:**
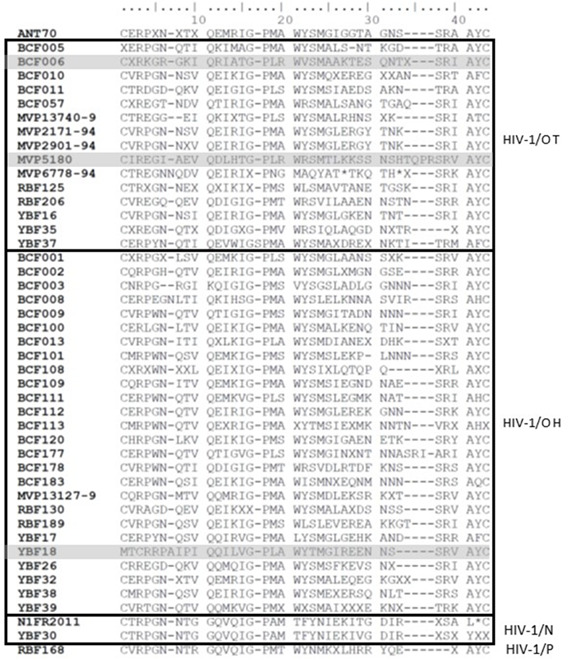
Alignment of V3 amino acid sequences from the HIV-1 non-M panel.

The hypervariability of the C2V3 region across and within each HIV-1 non-M group made it difficult to search for a genotypic correlation. However, we noticed that the MVC susceptible DM HIV-1/O strain YBF18 had a non-conservative genomic pattern in the first nine amino acids (MTCRRPA) (Fig. 4). There was no evident pattern specificity or mutation associated with the highest fold change (FC) nor with the DM tropism. Comparing the genetic sequence of the three groups of slopes, we noticed that three out of five strains presenting the highest slopes (<−2) harbored an insertion (asparagine N or alanine A) in position 7.

## DISCUSSION

The aims of the TROPI-CO study were to explore the *in vitro* susceptibility of a large panel of HIV-1 non-M strains to MVC, link these results to the R5, X4, or DM tropism determined by a phenotypic assay, and explore a potential genotypic pattern associated with phenotypic tropism or MVC susceptibility. Indeed, the existing tropism assays and genotype analysis algorithms are not suitable for non-M HIV-1 strains due to their high genetic variability, especially in the envelope region. Additionally, the limited number of individuals infected with those strains hinders the development of an accurate non-M-dedicated genotype algorithm.

The MVC susceptibilities for the HIV-1/M strains were consistent with what was expected from the identified tropism, validating our cellular assays for tropism and susceptibility determination. The large panel of HIV-1 non-M strains presented a global susceptibility to MVC, with a mean IC_50_ value of 1.23 nM, compared to group M, with an IC_50_ at 1.89 nM. Although a variability between culture methods can exist, these results are in accordance with the range of IC_50_ published by EMEA for O and M susceptible strains: 0.1–8.9 nM (15). In a previous study conducted on a limited number of HIV-1/O strains in a U87 human glioma cell-based assay, MVC susceptibility demonstrated a higher IC_50_ variability for HIV-1/O (ranging from 1 to 315 nM, mean at 51.2 nM) than for HIV-1/M (from 2 to 102 nM, mean at 32.4 nM) (1). In this particular *in vitro* study with non-primary cell lines, HIV-1/O seem less susceptible to MVC. A total of 15 strains were R5-tropic and 3 were DM-tropic, based on RT-activity quantification in the supernatant of R5_U87 or X4_U87 cultures, but without mention of the corresponding values of RT activity and IC_50_ (1). When comparing this study to our panel, we included an additional 12 HIV-1/O and had 29% of the strains in common. We can assume that the higher IC_50_ came from the DM viruses, as observed in our panel. The two strains with FC around 400 were DM-tropic. Our observation also showed that there was no complete correlation between MVC susceptibility and R5 tropism because one DM strain (YBF18) showed full susceptibility to MVC (MPI at 94.7% and FC < 1). In fact, the literature has reported an inefficiency of MVC with this tropism (16–18). It is possible that the DM tropism is not equally distributed among the strains’ sub-populations, with a higher proportion of R5 susceptible viruses in the YBF18 strain. This hypothesis still needs to be further explored, for example, by cell flow cytometric-directed analysis.

We also noticed that the amino acid composition of the V3 loop of YBF18 is very singular, with the presence of two consecutive basic amino acids (R = arginine) in the unique nine-amino-acid-long pattern (MTCRRPA). From the results obtained by Biscone et al. (19), we may also hypothesize that mutations in the co-receptor binding site of the YBF18 gp120 region may increase the MVC susceptibility. More globally, our results showed a wide range of MVC susceptibilities. This could be partly explained by the higher genetic diversity found in the HIV-1/O envelope than in group M variants (8). This diversity observed in the group O envelope may lead to discrepancies in virus interaction with the CCR5 coreceptor, both in the presence or absence of MVC inhibition. However, the genotypic specificities of HIV-1/O alone are insufficient to fully account for these various inhibition curves. In a previous phenotypic study of anti-gp41 enfuvirtide, Depatureaux et al. (5) demonstrated that the common N42D mutation, which is polymorphic and signature in HIV-1/O, does not correlate with the same phenotypic response across all the strains. A dedicated study utilizing directed mutagenesis could offer insights into the individual contributions of each mutation, within the genetic context of group O, shedding light on their roles in the phenotypic response to MVC.

Another critical issue about our culture method warrants attention. The use of PHA-stimulated peripheral blood mononuclear cells (PBMCs) may have introduced activated CD8+ T cells, thereby producing increased levels of CC-chemokines (such as CCL3, CCL4, and CCL5). This may have resulted in the inhibition of HIV-1 infection. Nevertheless, our approach gave satisfactory results in R5 infection (up to 7 Log10 viral titer in the non-MVC treated condition), even without depleting T cells from PBMCs. Our findings were also directly comparable to previous studies performed with the same protocol (7).

The absence of MVC-resistant strain in our HIV-1 non-M panel is underlined by the absence of X4 tropism (IC_50_ > 1,000 nM and MPI around 0%). As far as we know, there is no description of X4 HIV-1/O or other non-M strains in the literature (1, 3, 20), potentially meaning that the virus may use other coreceptors for the early steps of entry into the host cell. This could emphasize the potential role of antiretroviral therapy, including MVC, for those naturally highly divergent viruses. Regarding a deeper investigation into the MVC susceptibility, we noticed that a high IC_50_ value, and therefore a high FC, was not always associated with a weak MPI. Consequently, those two elements cannot completely characterize the susceptibility profile of the strain. In addition, concerning the strains with MPI > 90%, all were susceptible to MVC but presented different inhibition curve slopes, suggesting potential differences in therapeutic responses to MVC. That is why we propose to also consider the hill slope for additional parameters of susceptibility characterization, along with IC_50_/FC and MPI. For example, the HIV-1/N strain (N1FR2011) showed an FC at 40 but remained susceptible. Its inhibition curve showed a maximum decrease in the viral load between 10 and 100 nM concentrations, with a hill slope of −1.09, and therefore cannot be assigned to a resistant strain despite its high FC and intermediate MPI. In contrast, the susceptible strains MVP13740 and MVP6778-94 have quite similar IC_50_ at 0.79 and 0.67 nM, respectively, although the first strain shows a slower dynamic of inhibition (hills slope at 0.56) than the second strain (hill slope at 2.32). Thus, a potentially different therapeutic response could be expected for infected patients.

The hill slope information obtained from a standardized graphical representation of the inhibition parameters could therefore complement the other traditional measurements such as IC_50_, MPI, IC_90_, or IC_95_. While these metrics provide valuable information on the level of inhibition (IC) and the capacity of inhibition (MPI), the hill slope analysis offers insights into the dynamics of how effectively viral replication is inhibited as the drug concentration increases. This variation could suggest differences in the kinetics of drug action toward a specific strain. This proposition is in line with Jarantow et al.’s (21) discussion about standardization in biological assays with the best-fitting statistical model. The dose-response curves are based on a four-parameter logistic (4PL) equation also known as the hill equation. The steepness of the curve, reflected by the hill slope, can vary depending on the system under study. The 4PL model offers a robust framework for analyzing dose-response relationships, a crucial aspect when studying the efficacy of maraviroc *in vitro*. It also allows comparison between different dose-response curves, with the inherent variability of a PBMC assay, similar to the one we used. However, it also has some limitations. The assumption underlying the 4PL model is the symmetry between each part of the inflection point. In fact, this symmetry is not always true between the different assays, due to specific strain behavior during the phenotypic experiment. The complexity of the 4PL model requires careful interpretation of its parameters, as this could lead to biased conclusions.

However, the different inhibition slopes suggest different hypotheses concerning HIV-1 interactions with MVC. One hypothesis could be that MVC interacts with the HIV-1 CCR5 coreceptor directly. As it is the only antiretroviral drug with this mechanism of action, the subsequent inhibition of viral entry may contribute to the slope’s variability. The high genetic diversity of non-M HIV-1 could also explain the observed hill slopes. Indeed, a high genetic diversity exists in the V3 loop of non-M HIV-1. This genetic diversity could lead to varying strain interactions with co-receptors.

Altogether, these parameters could provide a more comprehensive understanding of the HIV strains’ susceptibility profiles.

To conclude, the phenotypic results obtained with 45 non-M HIV-1 strains confirm the critical importance of defining viral tropism before initiating MVC treatment in patients infected with HIV-1 non-M. Among the 45 HIV-1 non-M strains tested, 40 strains were susceptible to MVC compared to HIV-1 M strains. These 40 strains comprised either R5 or DM tropic viruses and presented high susceptibility to MVC treatment.

## MATERIALS AND METHODS

### Cells and virus stocks

Peripheral blood mononuclear cells were isolated at the Etablissement Français du Sang from three different healthy donors to avoid unique donor bias on type cells’ effects and extracted using Ficoll-Plaque Plus solution (GE Healthcare). PBMCs were re-suspended in RPMI-1640 medium (Lonza Bioscience) supplemented with 10% FBS (PAA Laboratories), 50 µg/mL gentamicin (Panpharma), and 0.2 µg/mL phytohemagglutinin (PHA) and grown at 37°C with 5% CO_2_. After 72 h, PBMCs were activated with interleukin-2 450 IU/mL (Proleukin, Novartis Pharma) and polybrene 2 µg/mL (Sigma-Aldrich) for at least 2 h before infection.

GHOST cells expressing CCR5 or CXCR4 were provided by the NIBSC. Those human osteosarcoma cells constitutively express CD4 receptor and the HIV coreceptor CCR5 or CXCR4. They also contain, as an HIV infection reporter gene, an HIV-2 long terminal repeat (LTR) sequence linked to a green fluorescent protein (GFP) gene. HIV infection induces viral Tat protein production, activates the HIV-2 LTR promoter, and generates GFP expression. Thus, HIV entry can be monitored by fluorescence intensity observation (1). The cells were cultured in DMEM with 10% FBS (PAA laboratories) and 1% penicillin and streptomycin (Sigma-Aldrich). In order to maintain the expression of the coreceptors, hygromycin 100 µg/mL (Invitrogen), puromycin 10 µg/mL (Sigma-Aldrich), and Geneticin G418 500 µg/mL (Invitrogen) were added in the maintenance medium, as recommended.

HIV-1 non-M strains were isolated from HIV-positive patients (cells or plasma samples) and cultivated for 3 weeks on PBMCs collected from healthy donors (RPMI, 10% FBS supplemented, gentamicin 50 µg/mL, at 37°C with 5% CO_2_). Half of the medium was replaced twice a week. During this period, the amount of virus was regularly quantified in the supernatant by measuring the activity of the reverse transcriptase (Lenti RT activity kit, Cavidi). Once the peak of activity of the viral enzyme was reached, the culture was stopped, and aliquots of viral supernatant were stored at −80°C.

Five HIV-1/M strains (BRU-HXB2, JR001, ARP1196, ARP1102, and ARP1129) with previous characterized tropism one HIV-1/M strain with R5, three with X4 tropism (ARP1196, BRU-HXB2, and JR001), and one DM tropism (ARP1129) were added as controls. These five strains were chosen as references for both drug phenotypic susceptibility and tropism assays. For this study, we analyzed 45 HIV-1 non-M clinical isolates: 42 HIV-1/O, 2 HIV-1/N, and 1 HIV-1/P, representative of HIV-1 genetic diversity as demonstrated with a phylogenetic tree based on V3 sequences (Fig. 1).

### Tropism assay

To determine the tropism of each strain, we used 2 × 10^4^ GHOST cells expressing co-receptor CCR5 or CXCR4 seeded in a 96-well plate, which were then incubated at 37°C, 5% CO_2_. After 12 h, to obtain cell confluence, 75 µL of HIV supernatant was added in triplicates (final volume of 200 µL) for 3 h, then the cells were washed with PBS before adding 200 µL of DMEM. On day 3, fluorescence from infected GHOST cells was checked by fluorescent microscopy to determine tropism. R5 tropism was characterized by fluorescent CCR5 GHOST cells (green) and non-fluorescent CXCR4 GHOST cells (colorless) and vice versa. If both cells turn out to express green fluorescence, a DM tropism is defined.

### Phenotypic assay and RNA extraction

The phenotypic assay was performed as previously described (22). Briefly, fresh PHA-stimulated PBMCs were infected by 100 TCID_50_/mL of HIV-1 supernatant for 2 h and then cultivated with five serial dilutions of increasing concentrations of MVC (0.1, 0.5, 2, 5, 10, and 100 nM) during 72 or 96 h. Each dilution was tested in quadruplicate in a 96-well plate. The quadruplicates were pooled and then RNA extraction was performed by the automated EZ1 advanced XL system (Qiagen) with EZ1 DSP Virus kit (Qiagen).

### qRT-PCR analyses

HIV-1/O RNA was quantified on a CFX96 Deep-Well (BioRad) by targeting integrase O thanks to primers and TaqMan probe: forward TCTATTACAGAGACAGCAGAGAYC, reverse CTACTGCTCCYTCACCTTTCC, and probe FAM-ACAGGAGYTGKGCCGGTCCTTTC Dark Quencher with RNA UltraSense One-Step Quantitative RT-PCR System (Invitrogen). The PCR was as follows: retro-transcription (RT) for 15 min at 50°C, denaturation for 2 min at 95°C followed by 50 cycles of cDNA amplification and denaturation for 15 s at 95°C and 30 s at 60°C. Genome equivalent concentrations were determined by extrapolation from a standard curve generated from serial dilutions of total HIV-1/O RNA of a known concentration.

HIV-1/N and M strains were quantified by targeting the LTR region using GENERIC HIV Charge Virale Kit (Biocentric) performed on CFX96 Deep-Well (Biorad) with the following program: RT for 10 min at 50°C and 5 min at 95°C, amplification of cDNA 50 cycles for 15 s at 95°C and 1 min at 60°C.

HIV-1/P strain was quantified using Xpert HIV-1 Viral Load Kit (Cepheid), which was proven as adapted to this strain from previous results of performance (23).

### Sequencing of the V3 loop region and phylogenetic analysis

Amplification of the C2V3 coding region of HIV-1/O was performed by PCR (Invitrogen Superscript III One step RT-PCR for long template kit). The PCR conditions were 30 min at 50°C, 2 min at 94°C, then 35 cycles of amplification (30 s at 94°C, 30 s at 55°C, and 2 min at 68°C) and finally 10 min at 68°C just before reaching 4°C for storage. A nested RT-PCR (QIAGEN HotStar Taq Master Kit 1,000 units) was then performed from 2 µL of the previous amplicon for 15 min at 95°C and then 35 cycles of amplification (30 s at 94°C, 30 s at 55°C, and 90 s at 72°C) to finish with 7 min at 72°C before storage at 4°C. The primers used are listed in Table 3. Sanger sequencing was performed on CEQ 8000 with the following program: 30 cycles (25 s at 96°C and 25 s at 50°C) followed by 4 min at 60°C and then 4°C using CEQ DTCS Quick Start kit. The sequences were analyzed and aligned using Genome Lab and MEGA 6 software to construct the phylogenetic tree by the neighbor-joining method (500 bootstraps, calculation with the two parameter Kimura method). The V3 sequences for the two HIV-1/N and HIV-1/P strains were extracted from sequences previously published in GenBank (JN572926, AJ006022, and GU111555, respectively).

**TABLE 3:** Primers used for the C2V3 region of HIV-1/O amplification and sequencing

PCR step	Sequence (5′−3′)	Fragment size (bp)
External	GGCTTTGMTAAYCCCATGTTTGA	1,533
	CCCATAGTGCTTCCTGCTGC	
Nested	ATTCCAATACACTATTGTGCTCCA	518
	AAAGAATTCTCCATGACAGTTAAA	
Sequencing	ATTCCAATACACTATTGTGCTCCA	518
	AAAGAATTCTCCATGACAGTTAAA	

## Statistical analysis

The analysis of concentrations of viral RNA in GE/mL was based on a qRT-PCR standardization to report the concentration from the cycle threshold.

The drug concentration that inhibits strain replication by 50% (IC_50_) was defined. The maximum percentage of inhibition corresponding to the point where the viral replication reaches its minimum under drug effect, by comparison to the point without drug, was also defined. These parameters were calculated with Microsoft Office Excel software. GraphPad Prism was used for drawing the graphs and calculating the hill slope after transformation of the concentration in decimal logarithm and normalization with 100% for the highest value and 0% for the lowest. Unpaired *t*-test (*P* < 0.05) was applied on the mean of our IC_50_ of HIV-1/M and IC_50_ of HIV-1/M strain reference.
